# A derivatization-enhanced detection strategy in mass spectrometry: analysis of 4-hydroxybenzoates and their metabolites after keratinocytes are exposed to UV radiation

**DOI:** 10.1038/srep39907

**Published:** 2017-01-06

**Authors:** Yi-Hsuan Lee, Ying-Chi Lin, Chia-Hsien Feng, Wei-Lung Tseng, Chi-Yu Lu

**Affiliations:** 1 Department of Biochemistry, College of Medicine, Kaohsiung Medical University, Kaohsiung 80708, Taiwan; 2 School of Pharmacy, College of Pharmacy, Kaohsiung Medical University, Kaohsiung 80708, Taiwan; 3 Department of Fragrance and Cosmetic Science, College of Pharmacy, Kaohsiung Medical University, Kaohsiung, 80708, Taiwan; 4 Department of Chemistry, College of Science, National Sun Yat-sen University, Kaohsiung 80424, Taiwan; 5 Research Center for Environmental Medicine, Kaohsiung Medical University, Kaohsiung 80708, Taiwan; 6 Institute of Medical Science and Technology, National Sun Yat-sen University, Kaohsiung 80424, Taiwan

## Abstract

4-Hydroxybenzoate is a phenolic derivative of alkyl benzoates and is a widely used preservative in cosmetic and pharmaceutical products. The presence of 4-hydroxybenzoates in the human body may result from the use of pharmaceutical and personal care products. These compounds are also known to exhibit estrogenic and genotoxic activities. The potential adverse effects of these compounds include endocrine disruption, oxidative and DNA damage, contact dermatitis, and allergic reactions. This study used two mass spectrometry methods that are applicable when using a derivatization-enhanced detection strategy (DEDS) to screen 4-hydroxybenzoates and their metabolites. Chemical derivatization was used to enhance the detection of these compounds. To evaluate the metabolic process triggered by UV radiation, human keratinocyte HaCaT cells treated with these 4-hydroxybenzoates were further exposed to UVA, UVB and UVC radiation. Metabolites transformed by human keratinocytes in the chemical derivatization procedure were identified by a nano ultra-performance liquid chromatographic system (nanoUPLC) coupled with LTQ Orbitrap. The experiments confirmed the feasibility of this method for identifying 4-hydroxybenzoate metabolites and for high-throughput screening of 4-hydroxybenzoate in commercial products (50 samples) by the DEDS.

Esters of 4-hydroxybenzoic acid (4-hydroxybenzoate), commonly known as parabens, are used as antimicrobial preservatives in cosmetics and pharmaceuticals. Parabens occur naturally in foods that have long-chain esters of 4-hydroxybenzoic acid, high antimicrobial activity, and low water solubility^1^. The presence of parabens in the human body mainly originates from the topical application of personal care products. Parabens are also known to have estrogenic and genotoxic activities^2^,^3^. Because of their widespread use as preservatives in various personal care products, cosmetics, pharmaceuticals, and food, parabens may be introduced to humans via many different environmental sources (including water, soil, sediment and sludge, air and dust, and biota)^3^,^4^.

Because these compounds are ubiquitous in the environment, their safety and toxicity should be clearly determined. The literature indicates that parabens may be a contributor to the obesity epidemic^5^ and may be markers of human breast cancer^6^,^7^,^8^,^9^. Parabens also have endocrine-disrupting effects^10^,^11^,^12^,^13^,^14^ and are known to induce oxidative and DNA damage^15^,^16^,^17^. By acting as haptens, parabens also cause contact dermatitis and allergic reactions^18^,^19^,^20^,^21^,^22^,^23^,^24^,^25^. Recent studies of pharmaceuticals and personal care products have shown that these compounds are pollutants^26^,^27^,^28^. Hence, controlling the use of these compounds is an important environmental issue.

The various methods used to detect parabens have been summarized in recent literature reviews^29^,^30^. Documented methods include liquid chromatography (LC), gas chromatography or capillary electrophoresis coupled with a detector (e.g., a flame ionization detector, a diode array detector, or a UV detector) or coupled with a mass spectrometer. For high throughput, matrix-assisted laser desorption ionization time-of-flight (MALDI-TOF) mass spectrometry (MS) is an effective technique for monitoring target analytes within a short time^31^,^32^. Hence, methods based on LC tandem MS and MALDI-TOF MS were developed for measuring parabens in various samples.

Recent studies have shown that metabolites produced by the combined effects of methyl paraben activated by sunlight irradiation and skin esterases can cause oxidative DNA damage^33^. The effects of UV light radiation on human keratinocyte HaCaT cells treated with these parabens were further evaluated by treatment with UVA, UVB and UVC. Cell line samples were composed of complicated components, and these biological samples were difficult to analyze by MS without sample preparation. After implementing the derivatization-enhanced detection strategy (DEDS), all paraben metabolites transformed by human keratinocytes were identified by LC coupled with LTQ Orbitrap. As pharmaceutical and cosmetic products are the major routes of human exposure to parabens^3^, this study also developed a simple MALDI-TOF MS method to screen for parabens in pharmaceutical and cosmetic products. After using a simple dilution method for sample preparation, detection sensitivity was increased by using chemical derivatization to label parabens. Experiments showed that the simple and rapidly performed method developed in this study is applicable for identifying paraben metabolites in human keratinocyte cells exposed to UV radiation and for screening for the presence of parabens in commercial pharmaceutical and cosmetic products.

## Results

Four common parabens in pharmaceuticals and cosmetics are methyl, ethyl, propyl and butyl esters of para-hydroxybenzoic acid. Because they act as haptens, parabens indirectly elicit immune reactions by interacting with larger endogenous proteins^25^,^34^. Hence, we designed two different experiments whereby the effects of paraben metabolites with and without exposure to UV light radiation were examined in human keratinocyte cells. Cell media contains complicated components, and these biological samples were difficult to analyze by MS without sample preparation.

**Figure 1:**
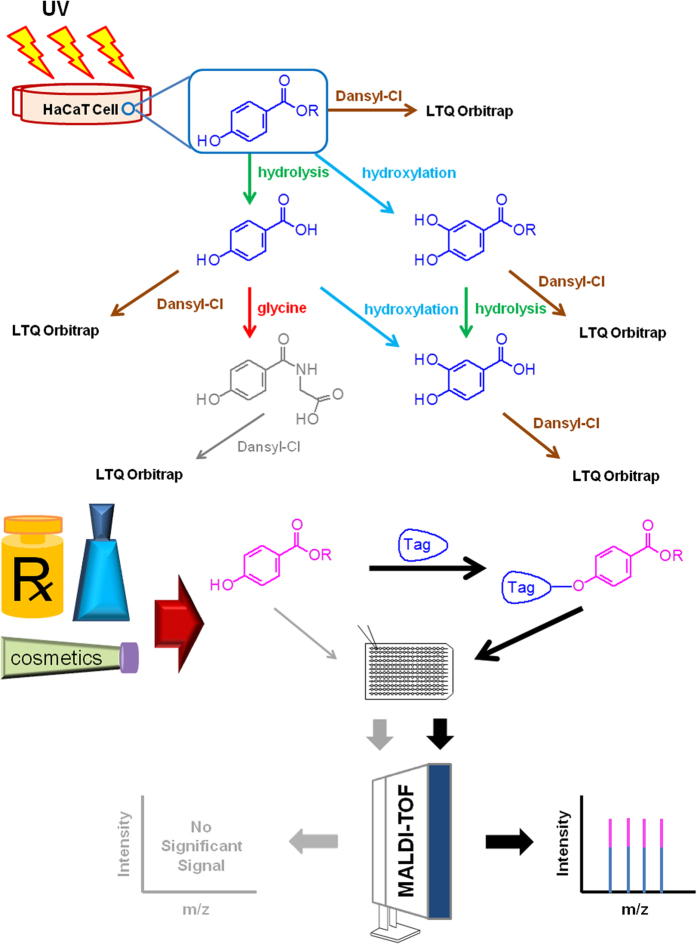
Block diagram of the procedure for using the proposed method for paraben analysis.

To improve the sensitivity of detecting parabens, we used a derivatization strategy to measure trace levels of parabens without the need for complicated sample preparation. Three sulfonyl chloride reagents were used to label parabens with their phenolic hydroxyl groups. The parameters of the derivatization protocol were then optimized, including the derivatizing reagent type, the derivatizing reagent concentration, the reaction solvent type, the base catalyst type, the base catalyst amount, the reaction time, the reaction temperature, the extraction solvent type, and the extraction solvent volume. Figure 1 shows a flowchart of the method.

### Optimization of the derivatization procedure

**Figure 2:**
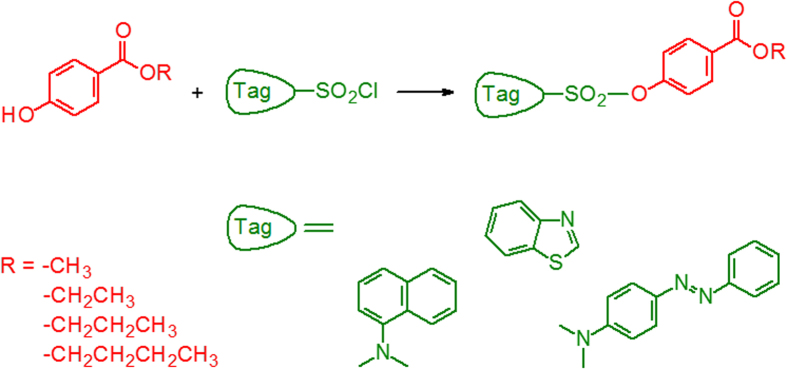
Simplified reaction scheme used for the chemical derivatization of parabens.

All factors that affected the formation of the paraben derivatives were studied. Figure 2 shows a simplified diagram of the reaction scheme for derivatizing parabens. Tags (such as 5-(dimethylamino)naphthalene, 4-dimethylaminoazobenzene and 1,3-benzothiazole) contain a sulfonyl chloride group that react with parabens in the presence of base catalysts at the micro-scale level.

The effects of different derivatizing reagents (Dansyl-Cl, Dabsyl-Cl and Batsyl-Cl) in a 0.5 mg/mL concentration on the formation of paraben derivatives were compared. The results presented in Supplemental Figure 1 show that using Dansyl-Cl as the reagent to label parabens resulted in the best signal for paraben analysis. The effects of different Dansyl-Cl concentrations (0.05–2 mg/mL) on paraben labeling were also compared. The data show that Dansyl-Cl at a 0.5 mg/mL concentration was optimal for the formation of paraben derivatives. Three different base catalysts, NaHCO_3_, Na_2_CO_3_ and NaOH at 200 mM, were used to compare their effects on the derivatization reaction. The results indicated that NaHCO_3_ was the optimal base catalyst for the formation of paraben derivatives. The results also indicated that following further comparisons of the effects of varying NaHCO_3_ concentrations (25–500 mM) on the derivatization reaction, NaHCO_3_ 200 mM was the optimal concentration. Further results revealed that based on comparisons of the effects of varying reaction times (5–30 min) on paraben derivatization, a reaction time of 20 min provided the best formation of Dansyl products. Comparisons of the effects of different reaction temperatures (30–80 °C) on Dansyl paraben derivatization indicated that 60 °C was the optimal reaction temperature for the production of derivatization compounds. Furthermore, the results showed that based on comparisons of the effects of the use of two different reaction solvents (acetone and acetonitrile) in the derivatization protocol, acetone was superior. Comparisons of the effects of three different water immiscible solvents (ethyl acetate, hexane and toluene) indicated that ethyl acetate was the best solvent in terms of extracting Dansyl parabens from the water layer to the organic layer. Finally, the results of tests of the effects of varying ethyl acetate volumes (20–60 μL) on the extraction of paraben derivatives from the water layer to the organic layer after derivatization demonstrated that 20 μL ethyl acetate was the optimal volume. All the optimal chemical derivatization procedures are shown in detail in Supplemental Figures 2–9. Supplemental Figure 10 shows the MS/MS spectra from nanoUPLC-LTQ Orbitrap used for the identification of paraben derivatives obtained by the optimal chemical derivatization procedure.

### Identification of parabens and paraben metabolites

**Figure 3:**
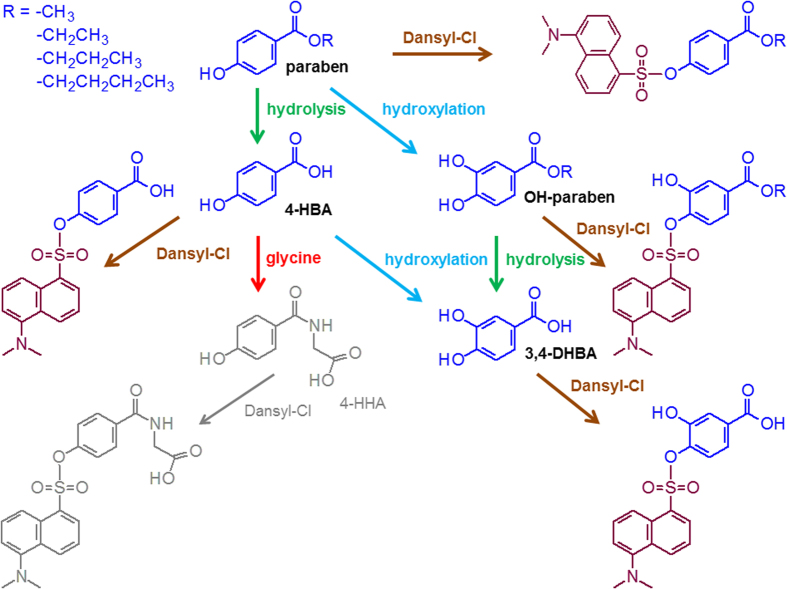
Biotransformation of parabens and their metabolites after the derivatization reaction. MP, EP, PP and BP are methyl, ethyl, propyl and butyl parabens, respectively; 4-HBA, 4-hydroxybenzoic acid; 3,4-DHBA, 3,4-dihydroxybenzoic acid; HHA, 4-hydroxyhippuric acid. Dansyl is the 5-(dimethylamino)naphthalene-1-sulfonyl group. HHA was not found in this study.

Parabens, which are esters of para-hydroxybenzoic acid, can produce several metabolites through various biotransformations. Because parabens are widely used as preservatives in cosmetics, sunlight can cause the photodegradation of parabens in cosmetic products applied to the skin. Hence, HaCaT cells (human keratinocyte cells) were experimentally treated with four different parabens. The cells were then exposed to UV light (UVA, UVB and UVC) for 2 hr to survey the effects of UV radiation on metabolic products. Hydrolysis causes cleavage of the ester bonds in parabens to form 4-hydroxybenzoic acid. Reaction with the amino group of glycine then forms the compound 4-hydroxyhippuric acid. A hydroxylation reaction can also biotransform 4-hydroxybenzoic acid into 3,4-hydroxybenzoic acid. In another metabolic process, parabens form OH-parabens by hydroxylation and then hydrolyze to produce 3,4-hydroxybenzoic acid. Figure 3 shows the potential metabolic pathways of parabens and the Dansyl products of parabens after the derivatization reaction.

This study revealed that in the cell media, four parabens, four hydroxylated parabens, and the compound 4-hydroxybenzoic acid reacted with Dansyl-Cl. The only compound found in HaCaT cells treated with methyl and ethyl parabens irradiated with UVC was 3,4-dihydroxybenzoic acid. The compound 4-hydroxyhippuric acid was not found. The chemical structures of the Dansyl parabens and their Dansyl metabolites were identified by LTQ Orbitrap. Tables 1 and 2 show the results.

The results in Tables 1 and 2 indicate that nanoUPLC-LTQ Orbitrap could detect more paraben metabolites than the MALDI-TOF MS method. Cell medium is a complicated biological sample, and it contains numerous compounds (such as inorganic salts, amino acids, vitamins, glucose, and proteins). The nanoUPLC-LTQ Orbitrap method contains a nanoUPLC column, and it has a greater ability to separate desired and undesired compounds into different zones within the nanoflow column. After separation by the nanoUPLC system, LTQ Orbitrap could detect the signal eluted from the nanospray source. The MALDI-TOF MS method does not have a separation function because this technique does not connect to any column. Hence, nanoUPLC-LTQ Orbitrap could detect more paraben metabolites than the MALDI-TOF MS method in a complicated biosample. No documented literature has indicated why the compound 3,4-DHBA was only detected in HaCaT cells exposed to methyl or ethyl paraben and UVC. We suppose that the side chains of methyl or ethyl paraben are shorter than those of propyl or butyl paraben. UVC contains a higher energy than UVB and UVA and thus may promote the biotransformation process. The short side-chain parabens may biotransform quickly under high-energy radiation. Perhaps the side chain length of paraben and the radiation energy are the reasons why 3,4-DHBA was detected only in HaCaT cells exposed to methyl or ethyl paraben and UVC.

### Analytical calibration, precision and accuracy

**Figure 4:**
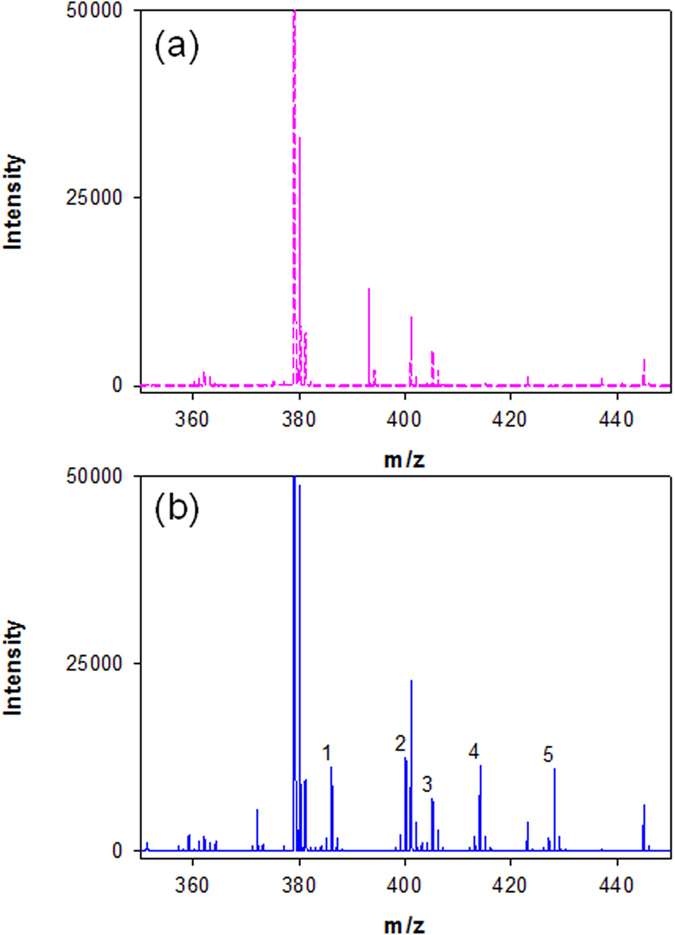
Typical mass spectra obtained in MALDI-TOF MS of (**a**) the reagent blank and (**b**) the paraben derivatives under optimal derivatization conditions. The [M + H]^+^ of signals 1, 2, 3, 4, and 5 are Dansyl-MP, Dansyl-EP, Dansyl-EP-d5 (IS), Dansyl-PP and Dansyl-BP at m/z 386.2, 400.2, 405.2, 414.2 and 428.2, respectively.

For high-throughput quantitative analysis of methyl, ethyl, propyl, and butyl parabens, the optimal protocol for the derivatization of parabens was performed over a linear range of 0.1–10 μg/mL by MALDI-TOF MS. For calibration curves, the peak area ratios of the Dansyl paraben derivatives to IS (Dansyl ethyl-d5 paraben derivative) were used as the y-axis and the paraben concentrations (μg/mL) were used as the x-axis. The paraben analysis results revealed high linearity with a coefficient of determination r^2^ ≥ 0.999 (n = 5). When this method was used for paraben analysis, the recovery was 91–103%, and the LOD was 0.025 μg/mL (tested by standard solution). The precision and accuracy of intra- and inter-day analyses of the parabens were tested at 0.25, 4.00 and 9.00 μg/mL concentrations. Table 3 shows that the RSD and RE were both below 9% for intra-day assays (n = 5) and inter-day assays (n = 5). The results in Table 3 also indicate that the proposed method utilized for paraben analysis had good precision and accuracy. The calibration curves for paraben quantitation are shown in Supplementary Table 1 and Figure 11. Figure 4 shows the MALDI-TOF MS spectra for paraben derivatives obtained by the optimal chemical derivatization procedure. Figure 4 also shows that the DEDS strategy is workable and suitable for the detection of parabens.

### Paraben analysis in human skin, pharmaceuticals and cosmetics

The proposed method was used to survey these compounds after applying cosmetic products to human skin. The amount of methyl paraben in the stratum corneum was determined by a cylinder sampling method in three volunteers after topical application of a cosmetic product containing methyl paraben. The quantitative analysis of methyl paraben was performed using MALDI-TOF MS. Table 4 shows that concentrations of 0.98 to 2.24 μg/cm^2^ were detectable after 60 min and that concentrations of 1.30 to 3.17 μg/cm^2^ were detectable after 120 min. The results indicate that methyl paraben could be retained in human stratum corneum and that this compound is a type of hapten. If the skin of cosmetics users is very sensitive and easily produces allergic reactions induced by methyl paraben, the users should select paraben-free cosmetic products.

In pharmaceutical and cosmetic products, parabens are used to prevent the degradation of unstable compounds. European Commission rules published in 2014 required manufacturers to reduce the concentration of these compounds. To safeguard human health, this study established a high-throughput method of detecting these parabens in pharmaceutical and cosmetic samples. Supplemental Tables 2–6 show that the method detected methyl, ethyl, propyl, and butyl parabens used as additives in pharmaceutical and cosmetic products. The data in Supplemental Tables 2–6 also show that methyl paraben is the preservative most commonly used to extend the shelf life of commercial products. All of these products contain at least one paraben. When consumers are hypersensitive to these preservatives, they should use these personal care products carefully.

## Discussion

Many methods can be used to detect parabens and these methods have been summarized in the literature^29^,^30^. These methods contain three commonalities: sample preparation before analysis, utilization of a column for sample separation, and utilization of a mobile phase (carrier gas or running buffer) for transporting analytes from the injector to the detector. Cosmetics contain many ingredients and are therefore very complicated samples. Complicated samples could shorten the life span of the column if the sample preparation is not well done. Before parabens can be analyzed using the documented methods, sample preparation is very important and necessary. Liquid-liquid extraction is a simple and easy method for sample preparation, but this strategy can produce substantial organic waste. Other liquid-based extraction methods for sample preparation (such as PLE, UAE and DLLME) are time consuming and labor intensive. In solid type clean-up methods for sample preparation (such as SPE, SPME and MSPD), SPE is widely used; however, cartridges for this process are expensive, and the procedure is time consuming and labor intensive. Furthermore, HPLC methods require a mobile phase for column separation, and this requirement leads to extra costs due to organic waste disposal. GC methods also require a carrier gas for column separation, and thus extra costs are incurred for the carrier gas. If the separation column is not cleaned completely before the next injection, contamination problems can occur.

In summary, the merits of the proposed method are as follows. (i) It minimizes the production of organic waste, the proposed method is a mobile phase-free method, and all analytical steps are at the micro-scale. (ii) The proposed method offers easy sample preparation, as the sample preparation procedures are simply dilution and centrifugation. (iii) The proposed method avoids contamination, as the reusable stainless target plate utilized in MALDI-TOF MS is easy to clean, with low contamination. (iv) Our method is high throughput, as the analytical time for MALDI-TOF MS is <1 min for one sample and the target plate can load 384 samples. (v) The proposed method is suitable for the analysis of parabens in many types of samples; for example, this method could be used for lotions, emulsions, creams, facial masks, pharmaceuticals, cell lines and human skin. (vi) The column and mobile phase (carrier gas or running buffer) cost is free.

This study developed a simple method of screening for parabens in human keratinocyte cells, commercial pharmaceuticals and cosmetic products. The results showed that the proposed method is effective for identifying paraben metabolites in human keratinocyte cells. After simple dilution of the cell media and commercial products, parabens were reacted with Dansyl-Cl. Dansyl parabens were then extracted by micro liquid-liquid extraction and detected by nanoUPLC coupled with LTQ Orbitrap and MALDI-TOF MS. As published papers have mentioned^35^,^36^, our results also indicated that dansylation was a suitable reaction for analysis of phenol-containing compounds by MS combined with an ESI or a MALDI interface. All analytical steps were designed to minimize the production of organic waste. Experiments showed that the proposed strategy is suitable for surveying parabens at the micro-scale level.

## Methods

### Materials and reagents

Methyl paraben, ethyl paraben, propyl paraben, butyl paraben, 5-(dimethylamino)naphthalene-1-sulfonyl chloride (Dansyl-Cl) trifluoroacetic acid (TFA), 4-dimethylaminoazobenzene-4′ sulfonyl chloride (Dabsyl-Cl), 1,3-benzothiazole-6-sulfonyl chloride (Batsyl-Cl), sparfloxacin, α-cyano 4-hydroxycinnamic acid (CHCA), sinapinic acid (SA), 2,5-dihydroxybenzoic acid (2,5-DHB), 2-mercaptobenzothiazole (2-MBT), 2-mercaptobenzoic acid (2-MBA) and 7-mercapto-4-methyl coumarin (7-MMC) were purchased from Sigma-Aldrich (St. Louis, MO, USA). Acetonitrile, acetone, dimethyl sulfoxide (DMSO), sodium bicarbonate (NaHCO_3_), sodium carbonate, (Na_2_CO_3_), sodium hydroxide (NaOH), formic acid, trifluoroacetic acid, ethyl acetate, hexane and toluene were purchased from Merck (Darmstadt, Germany). Ethyl-d5 paraben (internal standard, IS) was obtained from Toronto (Toronto, Ontario, Canada). All reagents were of analytical grade. Deionized water was obtained using a Millipore Milli-Q (Bedford, MA, USA) water purification system.

### Working solutions

Methyl paraben, ethyl paraben, propyl paraben, butyl paraben, ethyl-d5 paraben and sparfloxacin stock solutions (1 mg/mL) were prepared by dissolving the appropriate amounts of these chemicals in acetonitrile. A stock solution containing Dansyl-Cl, Dabsyl-Cl, and Batsyl-Cl (1 mg/mL) was prepared in acetone or acetonitrile. The NaHCO_3_, Na_2_CO_3_ and NaOH aqueous stock solutions (1 M) were prepared in water. Formic acid and trifluoroacetic acid (0.1%) were prepared in water. All matrices (10 mg/mL) used for MALDI-TOF MS analysis were prepared in acetonitrile: 0.1% trifluoroacetic acid = 50:50 (v/v).

### Derivatization of parabens

A 5-μL aliquot of standard paraben solution (mixed with 5 μL ethyl-d5 paraben at 10 μg/mL) in varying concentrations was transferred into Eppendorf tubes, and 10 μL Dansyl chloride (0.5 mg/mL) in acetone and 10 μL NaHCO_3_ (200 mM) in water were added. The solutions were incubated at 60 °C for 20 min. After reaction, 20 μL ethyl acetate was added and vortexed. After centrifugation at 10000 g for 1 min, 0.5 μL of the ethyl acetate layer was spotted on the target plate. Sparfloxacin (0.5 μL) and CHCA (0.5 μL) were further spotted on the target plate and mixed well for the MALDI-TOF MS analysis.

### Preparation of the paraben samples and identification of the paraben metabolites

Two different experiments were performed to test paraben metabolites with and without exposure to UV light radiation (8 W/cm^2^). Briefly, human keratinocyte HaCaT cells were cultured in Dulbecco modified Eagle’s medium (DMEM, Thermo Scientific, MA, USA) supplemented with 100 IU/mL penicillin, 100 μg/mL streptomycin, 2.5 μg/mL fungizone (Gibco, Grand Island, NY, USA), and 10% fetal bovine serum in a 5% CO_2_ incubator at 37 °C. The number of cells grown in each 10-cm tissue culture dish was counted when the cells reached 70–80% confluence. One experiment (without exposure to UV light radiation) used HaCaT cells treated with 100 μM (concentration in the culture medium) methyl paraben, ethyl paraben, propyl paraben or butyl paraben for 24 hr. The other experiment (with exposure to UV light radiation) used HaCaT cells treated with 100 μM (concentration in the culture medium) methyl paraben, ethyl paraben, propyl paraben or butyl paraben for 24 hr. Each sample was then exposed to UVC (254 nm), UVB (302 nm) and UVA (365 nm) for 2 hr. Finally, the cells were washed with phosphate-buffered saline, and medium samples were then collected and frozen at −80 °C until analysis.

### Sample preparation for paraben analysis of pharmaceuticals and cosmetics

The sample preparation procedure was simple and easy to perform. Liquid pharmaceutical and cosmetic samples (100 mg) were collected, and 1 mL acetonitrile was added. After sonication for 10 min, the sample solutions were centrifuged at 10000 rpm for 10 min. Aliquots (5 μL) of the sample solutions were transferred into Eppendorf tubes and subjected to the following derivatization protocol.

### Sample preparation for paraben analysis of human skin

To analyze parabens in human skin, a cylinder-sampling method was used^37^. Briefly, the forearm of the subject (age, 24 years; gender, 1 male and 2 female) was cleaned with water and dried. Then, 100 mg of lotion containing methyl paraben (0.03%) was applied to the forearm (area, 3.1 cm^2^). The open end of the cylinder was sealed with Parafilm, and the lotion was left on the skin. After 60 or 120 min, the lotion was transferred to a 1.5 mL centrifuge tube designated tube I. An alcohol pad was used to clean the forearm skin, and the pad was then collected in a tube designated tube II. Another glass cylinder was used to extract the methyl paraben from the stratum corneum with 0.5 mL ethanol, and the open end of the cylinder was sealed with Parafilm. After an extraction time of 5 min, the ethanol was transferred to a 1.5-mL centrifuge tube designated tube III. To examine the methyl paraben concentration in the stratum corneum after the application of cosmetics, a 5-μL aliquot from tube III was placed in a 1.5-mL centrifuge tube and evaporated until dry. After dissolving the residue in 30 μL water, the aqueous solution (5 μL) was used in further derivatization procedures. The experiments were approved by the Institutional Review Board of Kaohsiung Medical University Chung-Ho Memorial Hospital (KMUH-IRB-20130135). Written informed consent was obtained from all participants and the method was performed in accordance with the approved guidelines.

### Instrumentation

Parabens and their metabolites in cells were analyzed using a trapping column (Symmetry C18, 5 μm, 180 μm × 20 mm) and an analytical column (BEH C18, 1.7 μm, 75 μm × 150 mm) purchased from Waters (Milford, MA, USA). The nano ultra-performance liquid chromatographic system (nanoUPLC) had a nanospray ion source and was also manufactured by Waters. Parabens and their metabolite solutions (2 μL) were injected and then separated at a flow rate of 300 nL/min. Mobile phase A was 0.1% formic acid, and mobile phase B was acetonitrile (containing 0.1% formic acid). The gradient conditions were t = 0–1 min, hold B at 1%; t = 1–5 min, increase B from 12 to 100%; t = 5–45 min, hold B at 100%; and t = 45–60 min, decrease B from 100 to 1%. For structural identification, tandem mass spectrometry was performed with an LTQ Orbitrap Discovery hybrid Fourier Transform Mass Spectrometer (Thermo Fisher Scientific, Inc. Bremen, Germany). The LTQ Orbitrap was operated in positive ion mode with a nanospray source at a resolution of 30000. The voltages at the source, tube lens and capillary were set to 2.3 kV, 80 V and 28 V, respectively. The spray capillary temperature was set to 200 °C.

For paraben analysis, mass spectra were acquired in positive ion reflector mode by a MALDI-TOF MS system (model Autoflex III Smartbeam) equipped with a 355 nm Nd:YAG laser from Bruker Daltonics (Billerica, MA, USA). After spotting 0.5 μL of the sample solution on a ground target plate (Bruker Daltonics), 0.5 μL of the matrix solution (10 mg/mL) was added. Mass spectra were collected for the summing of 2000 laser shots, and the data processing was performed with FlexAnalysis software (Bruker Daltonics).

## Additional Information

**How to cite this article:** Lee, Y.-H. *et al*. A derivatization-enhanced detection strategy in mass spectrometry: analysis of 4-hydroxybenzoates and their metabolites after keratinocytes are exposed to UV radiation. *Sci. Rep.* **7**, 39907; doi: 10.1038/srep39907 (2017).

**Publisher's note:** Springer Nature remains neutral with regard to jurisdictional claims in published maps and institutional affiliations.

## Supplementary Material

Supplementary Information

## Figures and Tables

**Table 1:** Parabens and their metabolite derivatives identified by nanoUPLC-LTQ Orbitrap.

Compound^a^	Formula [M + H]^+^	Exact mass (Da)	Measured mass (Da)	Mass error (ppm)	MS/MS fragment
Dansyl-MP	C_20_H_20_NO_5_S	386.1057	386.1058	0.3	170.11, 171.06, 234.03, 354.09, 371.09
Dansyl-OH-MP	C_20_H_20_NO_6_S	402.1006	402.1008	0.5	120.05, 171.11, 237.11, 255.07, 356.17, 384.17
Dansyl-EP	C_21_H_22_NO_5_S	400.1213	400.1212	−0.2	170.09, 171.12, 234.09, 354.06, 372.14, 385.11
Dansyl-OH-EP	C_21_H_22_NO_6_S	416.1162	416.1161	−0.2	119.12, 170.10, 234.08, 252.10, 370.08
Dansyl-PP	C_22_H_24_NO_5_S	414.1370	414.1379	2.2	121.11, 170.12, 234.13, 354.14, 372.17
Dansyl-OH-PP	C_22_H_24_NO_6_S	430.1319	430.1315	−0.9	153.18, 196.16, 341.96, 370.13, 413.18
Dansyl-BP	C_23_H_26_NO_5_S	428.1526	428.1528	0.5	170.15, 234.12, 354.13, 372.17, 413.13
Dansyl-OH-BP	C_23_H_26_NO_6_S	444.1475	444.1481	1.4	174.22, 311.32, 354.05, 372.15, 426.15
Dansyl-4-HBA	C_19_H_18_NO_5_S	372.0900	372.0907	1.9	121.07, 171.08, 234.05, 354.17, 357.12
Dansyl-3,4-DHBA	C_19_H_18_NO_6_S	388.0849	388.0859	2.6	170.10, 234.22, 236.10, 356.17, 370.17

^a^MP,EP, PP and BP are methyl, ethyl, propyl and butyl parabens; 4-HBA, 4-hydroxybenzoic acid and 3,4-DHBA, 3,4-dihydroxybenzoic acid. Dansyl is the 5-(dimethylamino)naphthalene-1-sulfonyl group.

**Table 2:** Parabens and paraben metabolites in HaCaT cells with and without UV light treatment.

Compound^a^	UV Radiation			
	Control	UVC	UVB	UVA
MP treatment				
Dansyl-MP	V	V	V	V
Dansyl-OH-MP	V	V	V	V
Dansyl-4-HBA	V	V	V	V
Dansyl-3,4-DHBA	—	V	—	—
EP treatment				
Dansyl-EP	V	V	V	V
Dansyl-OH-EP	V	V	V	V
Dansyl-4-HBA	V	V	V	V
Dansyl-3,4-DHBA	—	V	—	—
PP treatment				
Dansyl-PP	V	V	V	V
Dansyl-OH-PP	V	V	V	V
Dansyl-4-HBA	V	V	V	V
Dansyl-3,4-DHBA	—	—	—	—
BP treatment				
Dansyl-BP	V	V	V	V
Dansyl-OH-BP	V	V	V	V
Dansyl-4-HBA	V	V	V	V
Dansyl-3,4-DHBA	—	—	—	—

^a^MP,EP, PP and BP are methyl, ethyl, propyl and butyl parabens; 4-HBA, 4-hydroxybenzoic acid; 3,4-DHBA, 3,4-dihydroxybenzoic acid. Dansyl is the 5-(dimethylamino)naphthalene-1-sulfonyl group. The symbols “V” and “—” mean detectable and undetectable, respectively.

**Table 3:** Precision and accuracy of paraben analysis by MALDI-TOF MS.

Concentration known (μg/mL)	Intra-day (n = 5)^a^			Inter-day (n = 5)^b^		
	Concentration found (μg/mL)	RSD^c^ (%)	RE^d^ (%)	Concentration found (μg/mL)	RSD^c^ (%)	RE^d^ (%)
Methyl paraben						
0.25	0.25 ± 0.01	4.00	0	0.25 ± 0.01	4.00	0
4.00	3.97 ± 0.09	2.27	−0.75	3.85 ± 0.04	1.04	−3.75
9.00	9.04 ± 0.12	1.33	0.44	8.96 ± 0.03	0.33	−0.44
Ethyl paraben						
0.25	0.23 ± 0.01	4.35	−8.00	0.24 ± 0.02	8.33	−4.00
4.00	3.82 ± 0.12	3.14	−4.50	3.87 ± 0.29	7.49	−3.25
9.00	8.92 ± 0.09	1.01	−0.89	8.90 ± 0.54	6.07	−1.11
Propyl paraben						
0.25	0.23 ± 0.01	4.35	−8.00	0.24 ± 0.02	8.33	−4.00
4.00	3.87 ± 0.16	4.13	−3.25	3.90 ± 0.23	5.90	−2.50
9.00	8.89 ± 0.24	2.70	−1.22	8.94 ± 0.45	5.03	−0.67
Butyl paraben						
0.25	0.24 ± 0.01	4.17	−4.00	0.23 ± 0.01	4.35	−8.00
4.00	3.86 ± 0.13	3.37	−3.50	3.95 ± 0.19	4.81	−1.25
9.00	8.95 ± 0.14	1.56	−0.56	9.27 ± 0.56	6.04	3.00

^a^Intra-day assay variance was calculated from the triplicate assay values on a single day.

^b^Inter-day assay variance was calculated from the triplicate assay values on five different days.

^c^RSD (%) = (SD/mean) x 100.

^d^RE (%) = [(Concentration found-Concentration known)/Concentration known] × 100.

**Table 4:** Methyl paraben concentration detected in the stratum corneum after application of cosmetic lotion (0.03%) from the triplicate assay values.

	Concentration (μg/cm^2^)		RSD (%)	
	1 hr	2 hr	1 hr	2 hr
Volunteer 1	0.98 ± 0.04	1.30 ± 0.03	4.1	2.3
Volunteer 2	1.94 ± 0.05	3.17 ± 0.07	2.6	2.2
Volunteer 3	2.24 ± 0.07	2.06 ± 0.11	3.1	5.3
Average	1.72	2.18		
