# Supplementary material for: A derivatization-enhanced detection strategy in mass spectrometry: analysis of 4-hydroxybenzoates and their metabolites after keratinocytes are exposed to UV radiation
Source: Sci Rep. 2017 Jan 6;7:39907. doi: 10.1038/srep39907 (PMC5216334; doi:10.1038/srep39907)
Supplement: Supplementary Information [file srep39907-s1.pdf]

Supplementary Information

A derivatization-enhanced detection strategy in mass spectrometry: analysis of 4-hydroxybenzoates and their metabolites after keratinocytes are exposed to UV radiation

Yi-Hsuan Lee [1], Ying-Chi Lin [2], Chia-Hsien Feng [3], Wei-Lung Tseng [4], Chi-Yu Lu [1, 5, 6, *]

[1] Department of Biochemistry, College of Medicine, Kaohsiung Medical University, Kaohsiung 80708, Taiwan

[2] School of Pharmacy, College of Pharmacy, Kaohsiung Medical University, Kaohsiung 80708, Taiwan

[3] Department of Fragrance and Cosmetic Science, College of Pharmacy, Kaohsiung Medical University, Kaohsiung, 80708, Taiwan

[4] Department of Chemistry, College of Science, National Sun Yat-sen University, Kaohsiung 80424, Taiwan

[5] Research Center for Environmental Medicine, Kaohsiung Medical University, Kaohsiung 80708, Taiwan

[6] Institute of Medical Science and Technology, National Sun Yat-sen University, Kaohsiung 80424, Taiwan

*Correspondence to:

Tel.: 886-7-3121101-2137-499; Fax: 886-7-322-3075

E-mail: cylu@kmu.edu.tw

Full postal address: 100, Shih-Chuan 1st Road, Kaohsiung, 80708, Taiwan

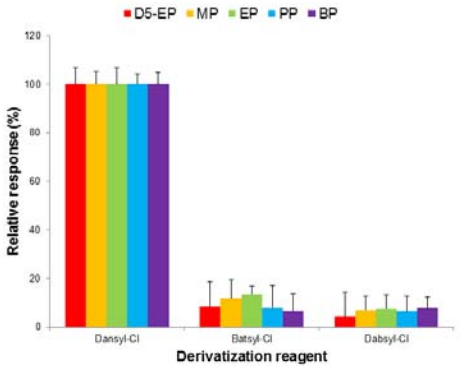

Figure S1. Effects of different derivatization reagents on the formation of paraben derivatives. MP, EP, PP, BP and D5-EP are the Dansyl derivatives of methyl, ethyl, propyl, butyl and ethyl-d5 parabens.

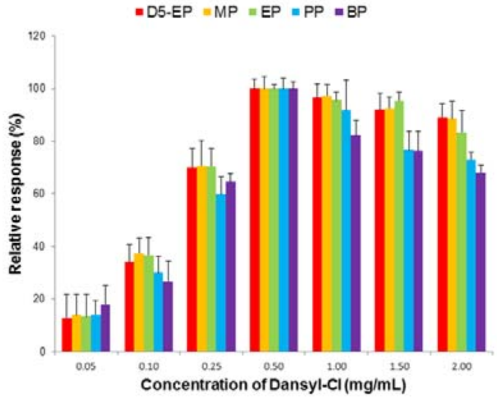

Figure S2. Effects of varying concentrations (0.25-10 mM) of Dansyl-Cl on the formation of paraben derivatives. The abbreviations are shown in Fig. S1.

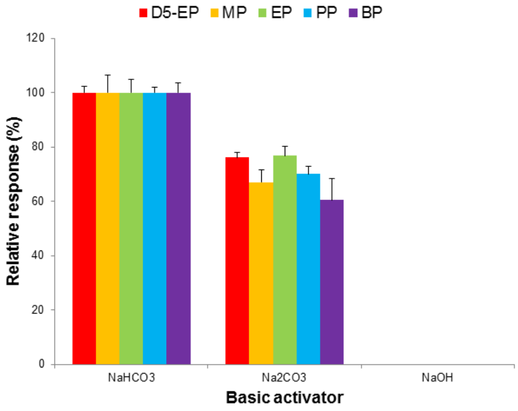

Figure S3. Effects of different basic activators ($NaHCO_3$, Na_2CO_3 and NaOH) on the formation of paraben derivatives. The abbreviations are shown in Fig. S1.

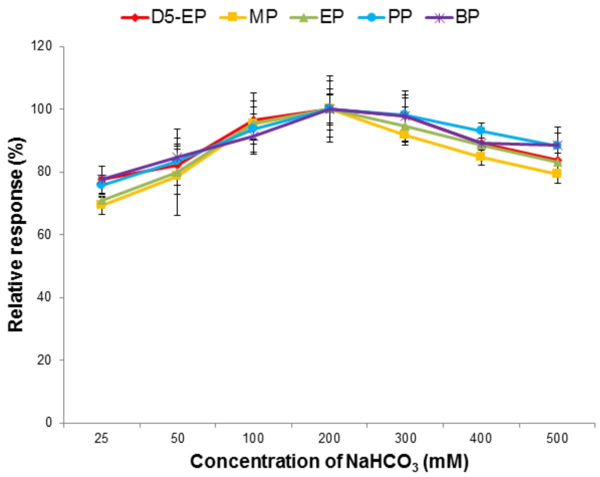

Figure S4. Effects of varying concentrations of $NaHCO_3$ (25-500 mM) on the formation of paraben derivatives. The abbreviations are shown in Fig. S1.

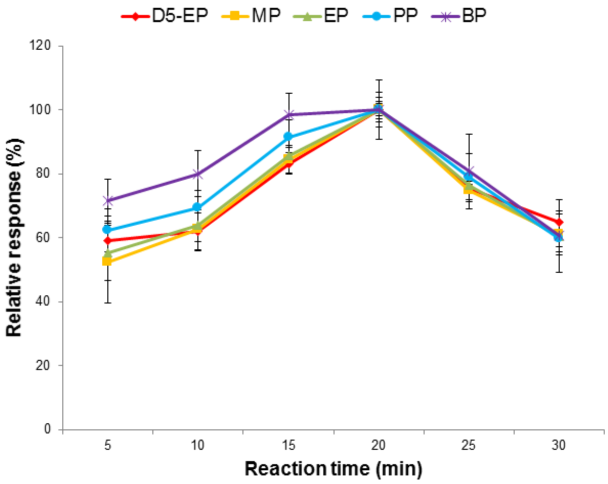

Figure S5. Effects of varying reaction times (5-30 min) on the formation of paraben derivatives. The abbreviations are shown in Fig. S1.

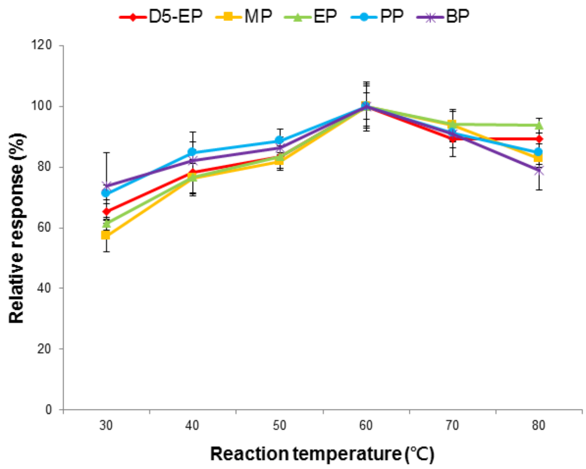

Figure S6. Effects of varying reaction temperatures (30-80 ℃) on the formation of paraben derivatives. The abbreviations are shown in Fig. S1.

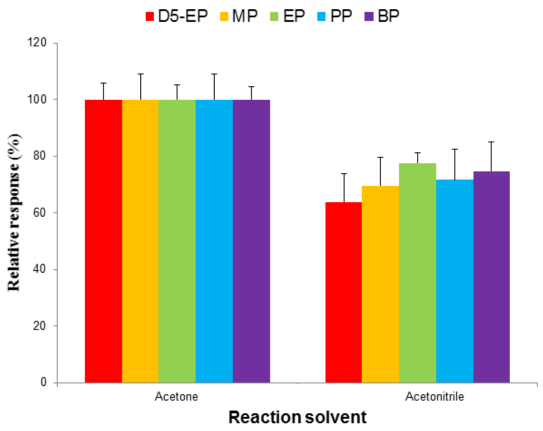

Figure S7. Effects of different reaction solvents (acetone and acetonitrile) on the formation of paraben derivatives. The abbreviations are shown in Fig. S1.

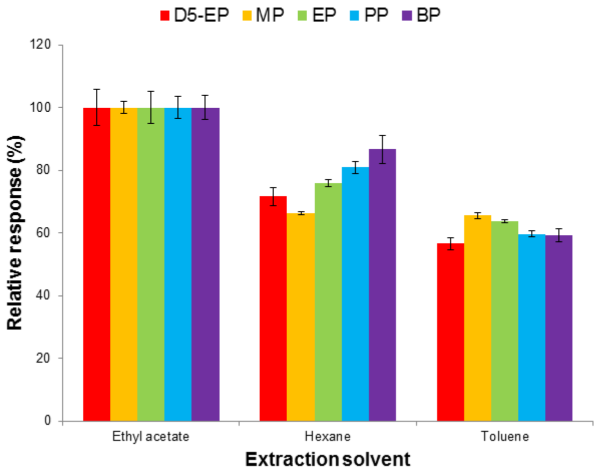

Figure S8. Effects of different extraction solvents (ethyl acetate, hexane and toluene) on the extraction of paraben derivatives after derivatization. The abbreviations are shown in Fig. S1.

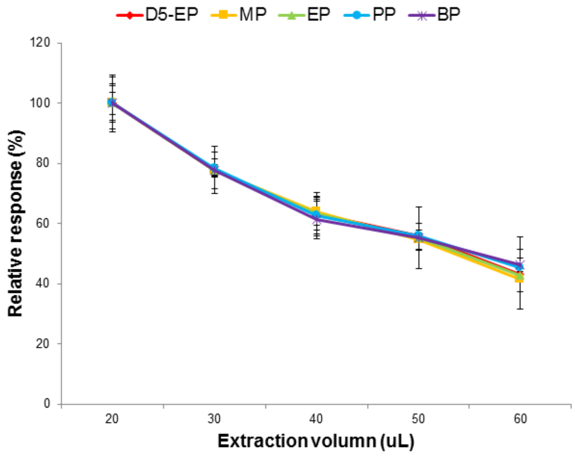

Figure S9. Effects of varying extraction volumes (20-60 μL) on the extraction of paraben derivatives after derivatization. The abbreviations are shown in Fig. S1.

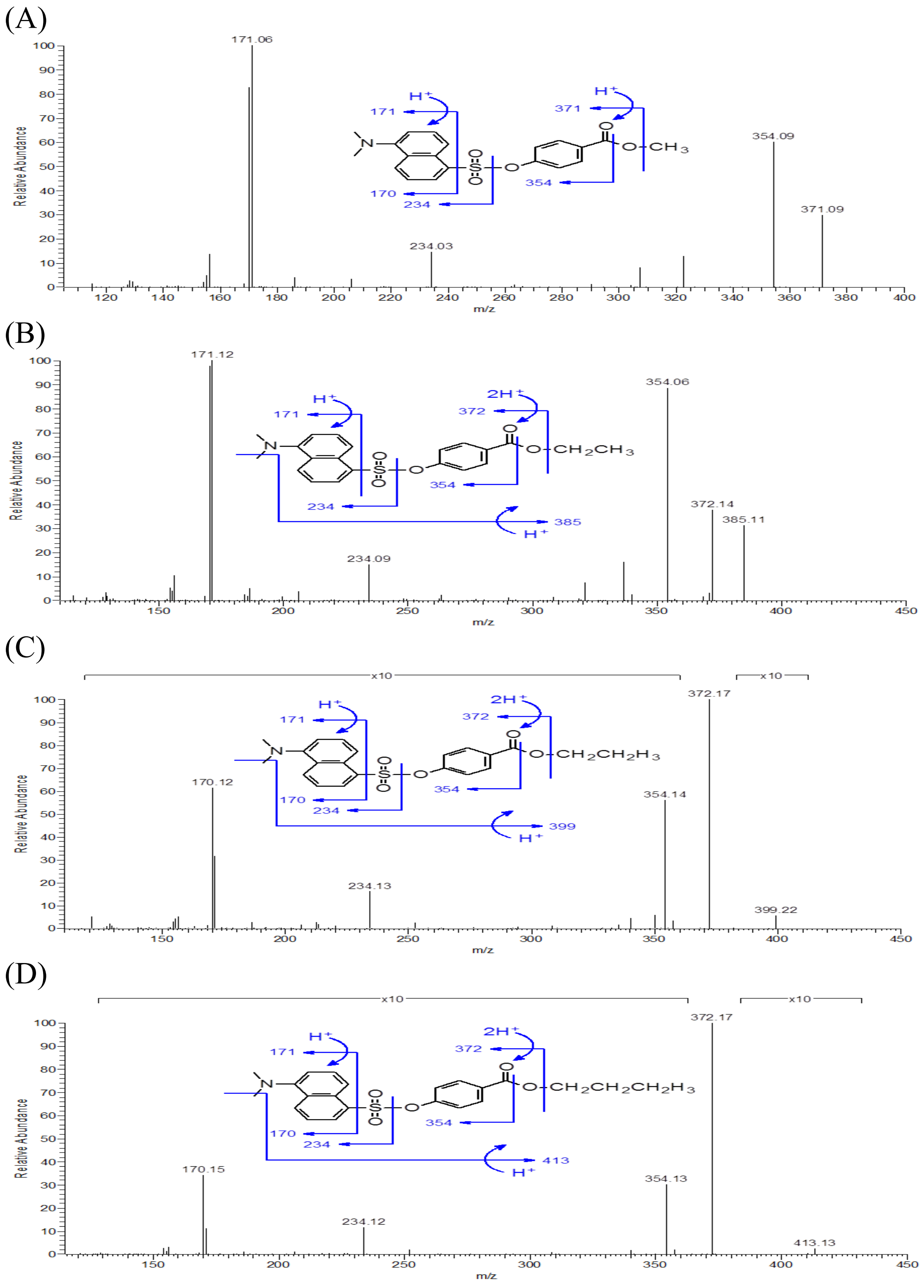

Figure S10. Typical nanoUPLC-MS/MS spectra and fragments obtained in LTQ Orbitrap paraben derivatives under optimal derivatization conditions; (A), (B), (C) and (D) are Dansyl-MP, Dansyl-EP, Dansyl-PP and Dansyl-BP.

Supplemental Table 1. Linear equation of parabens (n=5)

Analyte	Linear equation	r^2
Methyl paraben (MP)	y=(0.1475±0.0021)x-(0.0018±0.0001)	0.9993
Ethyl paraben (EP)	y=(0.1551±0.0007)x-(0.0032±0.0002)	0.9997
Propyl paraben (PP)	y=(0.1211±0.0012)x-(0.0039±0.0004)	0.9997
Butyl paraben (BP)	y=(0.1203±0.0020)x-(0.0130±0.0006)	0.9995

(A)

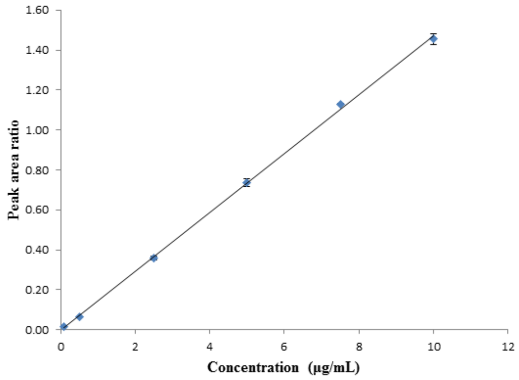

(B)

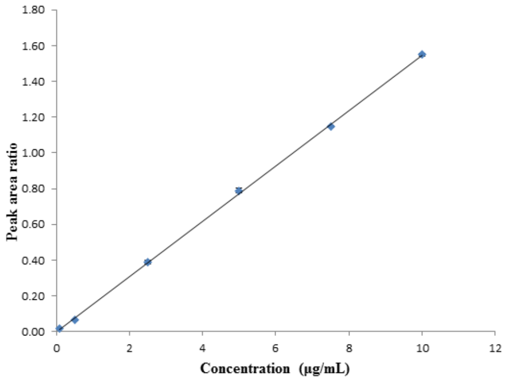

(C)

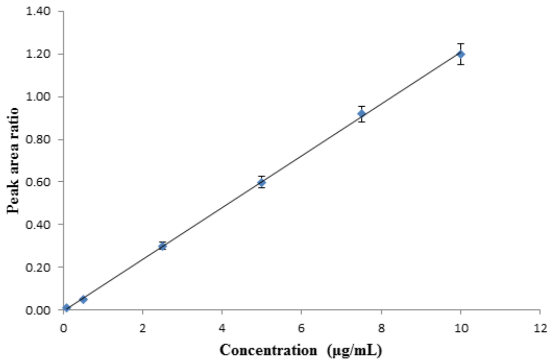

(D)

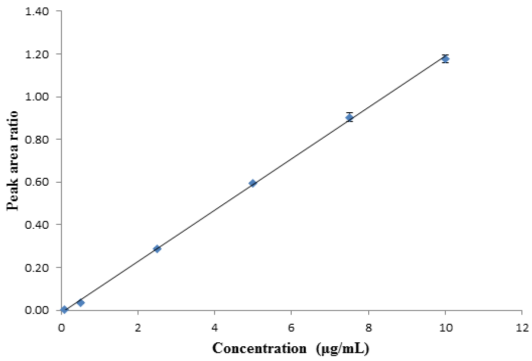

Figure S11. The calibration curves for (A), MP; (B), EP; (C), PP and (D) BP paraben quantitation.

Samples	Concentration (µg/mL)			
	**Methyl paraben**	**Ethyl paraben**	**Propyl paraben**	**Butyl paraben**
1	36.53±0.75	-	-	-
2	53.19±1.79	12.78±1.05	8.51±0.41	-
3	113.25±2.42	33.41±0.87	18.53±1.60	-
4	67.19±4.26	2.78±0.26	1.04±0.01	-
5	63.11±1.79	-	2.27±0.09	-
6	21.92±0.51	0.54±0.01	-	-
7	46.43±2.05	12.78±1.05	8.44±0.12	-
8	37.80±2.75	3.59±0.28	1.90±0.12	-
9	167.71±6.03	5.74±0.18	0.13±0.01	0.17±0.01
10	71.41±1.03	3.20±0.37	-	-
11	23.42±0.80	1.04±0.09	0.37±0.02	-
12	43.22±1.37	1.30±0.08	1.29±0.02	-

Supplemental Table 2. Determination of parabens in cosmetic lotions by MALDI-TOF MS from the triplicate assay values

Samples	Concentration (µg/mL)			
	**Methyl paraben**	**Ethyl paraben**	**Propyl paraben**	**Butyl paraben**
1	44.96±1.38	6.51±0.14	32.32±2.17	0.12±0.01
2	87.70±1.69	11.99±0.42	5.24±0.10	17.76±0.41
3	36.51±0.47	1.63±0.08	32.84±1.34	-
4	17.84±0.21	5.32±0.28	2.46±0.16	8.44±0.78
5	96.83±2.88	1.39±0.14	1.13±0.08	-
6	35.52±0.73	1.05±0.08	7.84±0.58	-
7	2.29±0.08	0.50±0.03	0.21±0.01	0.47±0.03
8	96.83±2.88	1.39±0.14	1.13±0.08	-
9	1.21±0.06	0.16±0.01	0.19±0.01	-
10	24.64±0.48	-	2.34±0.08	-

Supplemental Table 3. Determination of parabens in cosmetic emulsions by MALDI-TOF MS from the triplicate assay values

Samples	Concentration (μg/mL)			
	**Methyl paraben**	**Ethyl paraben**	**Propyl paraben**	**Butyl paraben**
1	64.28±2.12	35.85±2.57	14.39±0.91	37.62±0.92
2	42.61±1.12	11.11±0.58	4.97±0.34	30.21±1.97
3	1.02±0.04	99.95±1.95	55.49±1.41	-
4	54.20±1.44	2.46±0.09	1.34±0.08	1.89±0.04
5	52.93±1.00	1.06±0.09	17.28±1.15	-
6	9.55±0.17	0.11±0.01	0.14±0.01	-
7	3.89±0.32	2.70±0.18	19.60±1.32	-

Supplemental Table 4. Determination of parabens in cosmetic creams by MALDI-TOF MS from the triplicate assay values

Samples	Concentration (μg/mL)			
	**Methyl paraben**	**Ethyl paraben**	**Propyl paraben**	**Butyl paraben**
1	51.90±1.07	2.15±0.07	1.11±0.08	-
2	43.58±2.54	4.49±0.05	0.79±0.05	-
3	33.61±1.50	1.61±0.14	0.85±0.02	-
4	24.72±1.61	1.96±0.23	0.85±0.02	-
5	14.63±1.19	-	-	-
6	1.57±0.09	5.57±0.20	1.53±0.12	-
7	16.95±0.80	0.12±0.01	-	-
8	17.92±0.79	-	-	-
9	4.28±0.23	-	-	-
10	1.31±0.06	1.19±0.03	0.77±0.02	1.01±0.02

Supplemental Table 5. Determination of parabens in cosmetic facial masks by MALDI-TOF MS from the triplicate assay values

Samples	Concentration (μg/mL)			
	**Methyl paraben**	**Ethyl paraben**	**Propyl paraben**	**Butyl paraben**
1	39.76±1.17	0.87±0.05	-	-
2	225.50±16.64	6.05±0.17	7.49±0.26	-
3	39.69±0.60	-	-	-
4	48.69±0.64	-	-	-
5	10.07±0.36	0.50±0.04	3.30±0.12	-
6	2.02±0.04	-	0.76±0.02	-
7	34.90±1.97	-	-	-
8	-	14.81±0.49	-	-
9	-	1.62±0.02	-	0.67±0.04
10	119.79±7.66			
11	49.27±1.99	1.14±0.08		

Supplemental Table 6. Determination of parabens in pharmaceuticals by MALDI-TOF MS from the triplicate assay values
